# Low Hematocrit Is a Strong Predictor of Poor Prognosis in Lung Cancer Patients

**DOI:** 10.1155/2018/6804938

**Published:** 2018-10-17

**Authors:** Xin Zhang, Fengmin Zhang, Wenbo Qiao, Xiansheng Zhang, Zhefeng Zhao, Mingqi Li

**Affiliations:** ^1^Department of Radiotherapy, Harbin Medical University Cancer Hospital, Harbin 150001, Heilongjiang, China; ^2^Department of Microbiology, College of Basic Medical Science, Harbin Medical University, Harbin 150001, Heilongjiang, China; ^3^Heilongjiang Institute of Dermatology, Harbin 150001, Heilongjiang, China; ^4^Department of Neurosurgery, Second Affiliated Hospital of Harbin Medical University, Harbin 150001, Heilongjiang, China; ^5^Department of Surgery, Harbin Medical University Cancer Hospital, Harbin 150001, Heilongjiang, China

## Abstract

Morbidity and mortality of lung cancer rank first in China and worldwide. Thus, noninvasive prognostic biomarkers are critical for clinicians to perform risk assessment in lung cancer patients prior to or during the course of treatment. In this study, we evaluated the prognostic value of preoperative hematocrit (HCT) count reduction on the long-term survival of lung cancer patients undergoing pneumonectomy and analyzed the correlation between reduced HCT counts and patients' clinicopathological features. A total of 1022 patients who underwent surgical treatment in Harbin Medical University Cancer Hospital, China, from February 2006 to December 2013, were enrolled in this study. The association between the clinicopathologic variables and HCT were evaluated by Mann-Whitney U-test and chi-square test, respectively. Survival curves were generated using Kaplan-Meier estimates, and differences between the curves were analyzed by a log-rank test. Multivariable analysis showed that high HCT (P < 0.001, HR: 0.595, 95% CI: 0.458–0.774) was favorable for patients' survival. Low HCT patients presented shorter mean months of OS than that of high HCT patients (P < 0.001). Male adenocarcinoma carcinoma patients with lower body mass index (BMI) and advanced tumor stage were more likely to observe low HCT. We identified for the first time reduced preoperative HCT count as an independent risk factor leading to poor prognosis in lung cancer patients who underwent surgical treatment.

## 1. Introduction

Lung cancer is the most common malignancy worldwide. In the past three years, the morbidity and mortality of lung cancer have been increasing with unprecedented speed and ranking first in all cancers [1]. In 2015, the number of newly diagnosed lung cancer cases in China reached about 730 thousand and the mortality 610 thousand [2]. As generally acknowledged, it is important to accurately predict the prognosis of a patient before surgery for appropriate management of patients. Thus, biomarkers are critical for clinicians to perform risk assessment in lung cancer patients prior to or during the course of treatment.

Studies have reported that lung cancer patients with increased peripheral blood leukocytes showed increased mortality [3–5], and the number of lymphocytes and their abnormal functions were also indicators of prognosis [4, 5]. HCT is one of the erythrocyte parameters. However, there have seldom been reports to show that HCT can be adopted as a screening index or a treatment effect predictor of certain diseases [6]. No reports showed that it can be used in lung cancer cases, either.

In this study, we evaluated the prognostic value of preoperative HCT count reduction on the long-term survival of lung cancer patients undergoing pneumonectomy. In addition, the correlation between reduced HCT counts and patients' clinicopathological features was analyzed.

## 2. Materials and Methods

### 2.1. Study Population

A total of 1022 patients who underwent surgical treatment in Harbin Medical University Cancer Hospital, China, from February 2006 to December 2013 were enrolled in this study. The enrolled patients received routine chest CT, head CT, bone nuclide imaging, and liver and adrenal gland ultrasonography to evaluate the preoperative tumor staging and received pulmonary function and heart color ultrasonography to evaluate the heart and lung function. All enrolled patients were diagnosed through pathological diagnosis. For most lung cancer patients, standard surgical procedure of pulmonary lobectomy and node dissection was performed. Excluding criteria are preoperative neoadjuvant radio (chemo-) therapy, intraoperative or postoperative pathologic exclusion of lung cancer, and patients with lung cancer combined with other cancer. This study was approved by the Ethics Committee of Harbin Medical University, Harbin, China. Written informed consent was obtained from all the participants. In our study, the lung cancer TNM staging system was used, which was revised by the International Association for the Study of Lung Cancer (IASLC) and adopted by the AJCC (8th edition, 2017) [7]. The TNM staging system applying to both NSCLC and SCLC based on studies showed that the various stage designation presented prognostic significance in both diseases [8].

### 2.2. Follow-Up

We carried out the follow-up by calling or visiting the patients or their relatives to assess their medical records. The first follow-up performed after 1 month hospital discharge, when the patients were recommended to return to the hospital for routine postoperative examination. The second follow-up was carried out at the end of the third month after surgery. After that, the patients were followed up once every 3 months for the first year, then every 6 months for the second year and third year, and once for the fourth and fifth year. If a patient died during the follow-up, the information of the exact date and cause of death would be acquired. The last follow-up was in July 2016. The surviving patients at the end of the study were examined.

### 2.3. Clinical Data Collection

The following data of each patient was collected from the Hospital Episodes Statistics with permission of the hospital's ethnic committee: sex, age at the surgery, height, weight, smoking history, tumor size, pathological type, lymph node metastasis, staging, and survival time, etc. The other laboratory data collected included red blood cell (RBC) count, mean corpuscular hemoglobin (MCH), hemoglobin (Hb), white blood cell (WBC), prothrombin time (PT), hematocrit (HCT), and mean corpuscular volume (MCV). LH750 Beckman Coulter (Florida, Miami, USA) was used to measure blood cell counts.

If multiple laboratory tests were carried out, only the data from the last examination before surgery would be analyzed. The postoperative count was specified to the examination after 1 month of the surgery. According to the diagnostic criteria of the hospital, the above biochemical variables were divided into 3 groups: increased group (above the upper limitation of the normal range), normal group (within the normal range, if there was a range for laboratory parameters in clinical practice), and decreased group (less than the lowest limitation of normal range).

### 2.4. Statistical Analysis

The association between the clinicopathologic variables and HCT were evaluated by Mann-Whitney U-test and chi-square test, respectively. Cox univariate and multivariate analyses were performed to identify the independent factors relevant to patients' survival. Survival curves were generated using Kaplan-Meier estimates, and differences between the curves were analyzed by a log-rank test. All analyses were conducted using SPSS 21.0 (Xishu software (Shanghai) company). P values < 0.05 were considered statistical significance.

## 3. Results

### 3.1. Baseline Characteristics and Follow-Up Information of Lung Cancer Patients

We collected the preoperative clinicopathologic data and follow-up information of 1022 patients with lung cancer in this study. The baseline characteristics of these patients were summarized in Table 1. The study cohort included 590 men (57.7%) and 432 women (42.3%), with a median age of 59 years. All patients were followed up and the median follow-up time was 24.7 months (range, 1–114 months). At the time of final follow-up, there were 782 patients alive and the 1, 3, and 5 year overall survival (OS) rates were 92%, 74%, and 66%, respectively.

### 3.2. Survival Analysis for the Risk Factors Related to OS of Lung Cancer Patients

To investigate the risk factors for poor prognosis after surgical treatment, the preoperative HCT, RBC, Hb, MCV, and MCH as well as the other 6 potential clinicopathological characteristics were analyzed by univariate analysis. As shown in Table 2 HCT (P < 0.001), RBC (P = 0.001), Hb (P = 0.013), and tumor stage (P < 0.001) were identified as candidate risk factors for patients' prognosis. Furthermore, multivariable analysis showed that advanced tumor stage (tumor stage II: P < 0.001, HR: 1.969, 95% CI: 1.468-2.460; tumor stage III: P < 0.001, HR: 2.584, 95% CI: 1.866-3.578) was an independent risk factor for poor prognosis in patients with lung cancer, and high HCT (P < 0.001, HR: 0.595, 95% CI: 0.458–0.774) was favorable for patients' survival (Table 3).

**Figure 1:**
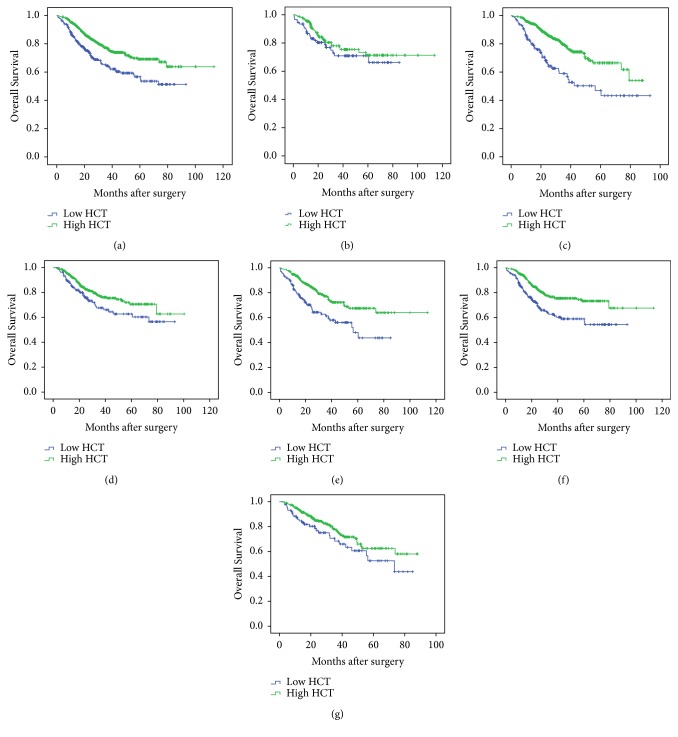
**Prognostic value of the preoperative HCT in patients with lung cancer.** (a) All patients with low HCT (n = 269) and high HCT (n = 753) (*P* < 0.001). (b) Squamous cell lung carcinoma patients with low HCT (n = 92) and high HCT (n = 177) (*P* = 0.281). (c) Lung adenocarcinoma patients with low HCT (n = 131) and high HCT (n = 462) (*P ***< **0.001). (d) Younger patients with low HCT (n = 135) and high HCT (n = 429) (*P* = 0.030). (e) Older patients with low HCT (n = 134), and high HCT (n = 324) (*P* < 0.001). (f) Male patients with low HCT (n = 181) and high HCT (n = 409) (*P* < 0.001). (g) Female patients with low HCT (n = 88) and high HCT (n = 344) (*P* = 0.066).

To the best of our knowledge, this was the first study to report the prognostic value of HCT in lung cancer patients. To further access the predicating value of HCT, we used the Kaplan-Meier method and log-rank tests. As the Kaplan-Meier survival curve shown in Figure 1(a), low HCT patients presented shorter mean months of OS than high HCT patients (P < 0.001). The 1-, 3-, 5-year OS rates were 85%, 64%, and 56% in low HCT group. The respective OS rates in high group were 94%, 77%, and 69%.

Next, to prove the universal applicability of HCT as a valuable prognostic parameter, the cohort was adjusted by histologic type. Patients were divided into lung adenocarcinoma patients and squamous cell carcinoma patients, and the results showed that HCT had a predictive prognostic value for lung adenocarcinoma patients (Figure 1(c)) and no such value for lung squamous cell carcinoma patients (Figure 1(b)).

Kaplan-Meier curve analysis confirmed that, among male patients, the 1-, 3-, 5-year OS rates were 85%, 62%, and 58% in low HCT group and 94%, 77%, and 73% in high HCT group; low preoperative HCT indicated poor prognosis (P < 0.001). However, this result was not obtained in female patients (P = 0.066). According to their ages at the moment of surgery, the patients were divided into 2 groups using age of 60 years old as a cutoff set, while, among a subfraction of patients under 60 years old, the 1-, 3-, 5-year OS rates were 88%, 68%, and 63% in low HCT group patients, which were statistically lower than 94%, 77%, and 71% in high HCT group (P = 0.030). Similar results were also obtained in those patients > 60 years old; low HCT group patients have shorter OS than high HCT group (P < 0.001) (Figures 1(d)–1(g)).

### 3.3. Impact of Preoperative and Postoperative HCT on the Prognosis of Lung Cancer Patients

**Figure 2:**
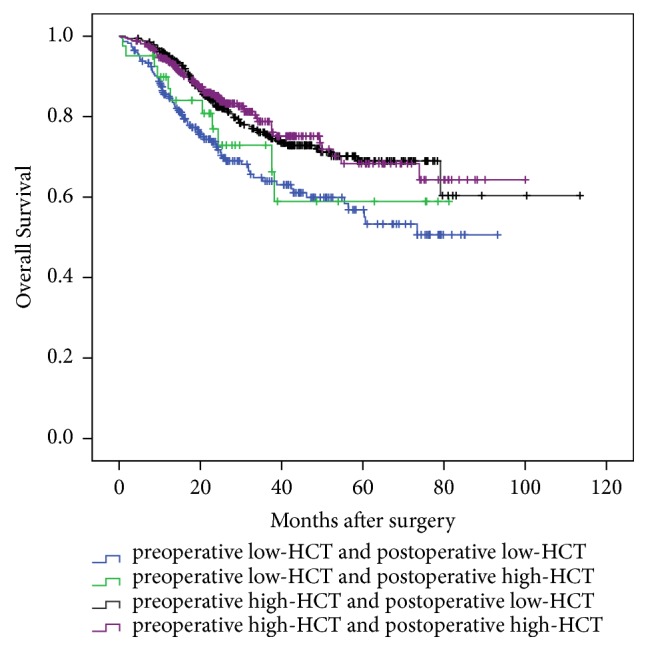
Comparison of overall survival among 4 subgroups of lung adenocarcinoma cancer patients.

As preoperative HCT could potentially be used as a predictor of the OS in lung adenocarcinoma cancer patients who underwent surgery, it was worthwhile to take the influence of postoperative HCT into consideration too. When preoperative HCT was combined, patients were stratified into 4 subgroups. The results were plotted in Figure 2. Preoperative HCT has a greater effect on the prognosis of patients with lung cancer. The prognosis of patients with high preoperative HCT is better while that of patients with low HCT is worse. Postoperative HCT has less effect in predicting patients' survival. It seemed that preoperative HCT was more usable in predicting patients' survival.

### 3.4. Comparisons of Characteristics between Patients with Low and High HCT

The characteristics of two groups were summarized. There was statistically significant association between two HCT groups in sex (P < 0.001), tumor staging (P = 0.011), BMI (P < 0.001), and smoking (P < 0.001). Multivariate analysis showed that sex, BMI, and tumor stage, influenced HCT, indicating that male adenocarcinoma carcinoma patients with lower BMI and advanced tumor stage were more likely to observe low HCT.

## 4. Discussion

Currently, there is no unanimous evaluation standard of prognosis of lung cancer patients, as a variety of factors can affect the patients' prognosis. In this study, through Cox univariate analysis, HCT, RBC, Hb, and tumor stage were identified as the candidate risk factors for patients' prognosis. Further, multivariate analysis showed that advanced tumor stage was an independent risk factor for poor prognosis in lung cancer patients, and high HCT was favorable for patients' survival (Table 3).

Some of these factors were in coherence with other studies [4, 9–11], which indicated the quality of our current follow-up study cohort. Interestingly, as the Kaplan-Meier survival curve shown in Figure 1(a), patients with low HCT presented shorter mean months of OS than patients with high HCT. We identified for the first time the reduced preoperative HCT count as an independent risk factor leading to poor prognosis in lung cancer patients who underwent surgical treatment. Furthermore, our data clearly demonstrated that HCT was negatively correlated with patients' tumor stage, which was a well-known prognostic measure for predicting the outcome of lung cancer patients who underwent surgical resection. Thus, it was not surprising to find that patients with lower preoperative HCT had shorter survival time, just as patients with higher tumor stage. In addition, since HCT can be examined by routine blood test, it might be adopted as a new and conditional marker to estimate the general condition of patients and predict the mortality risk of lung cancer patients who would undergo surgery.

It was commonly recognized that certain clinicopathologic variables could impact the OS rate of lung cancer patients who underwent surgical treatment. At the moment, we cannot provide a mechanistic explanation why a patient's preoperative HCT count linked to his/her postsurgery survival chance. An article recently published pointed out that the lung was a complex organ that could regenerate after major injuries, a major organ of platelet production with potential of being a contributor to blood generation, and a reservoir for resident megakaryocytes and hematopoietic progenitor cells [12].

In order to obtain some clues, we explored the relationship between reduced preoperative HCT with those demographic and clinical features known to be of impact on patients' postoperative survival rate. We found that male patients with lower BMI, adenocarcinoma carcinoma and advanced tumor stage were more likely to observe low -HCT.

Some studies have suggested that low HCT is associated with patients' loss of appetite. Nutritional status was an important predictor of postoperative death and complications in patients with lung cancer [13]. There have been articles pointing out that Low BMI before surgery affected surgical outcomes in the negative way for patients with non-small-cell lung cancer [14, 15].

Our findings also showed that low HCT was in association with lower BMI, which was related to poor prognosis. Red cells were associated with the immune status of the body [16]. Some studies concluded that lung cancer patients with anemia had reduced immune function [17], which was more obvious in late stage (IV-stage) lung cancer. Our findings identified that low HCT was an independent risk factor for prognosis in patients with early and midterm (II-stage and IIII-stage) lung cancer. As this study showed that low HCT in the early and midstage lung cancer affected prognosis, treating patients with anemia before surgery with iron supplements, folic acid, and vitamin B12 could decrease the anemia of majority of patients, and such treatment was very necessary [13, 18].

We must acknowledge that there are some limitations in this study. During the course of collecting cases, we do not consider the impact of surgical procedure and standardized treatment after surgery (such as whether to accept chemotherapy and targeted therapy, etc.) on the survival. Additionally, this study is a retrospective study; large, prospective studies will be necessary to determine.

## 5. Conclusions

We identified for the first time reduced preoperative HCT count as an independent risk factor leading to poor prognosis in lung cancer patients who underwent surgical treatment.

## Figures and Tables

**Table 1:** Baseline characteristics of the 1022 patients.

**Clinical feature**	**n**	**Percentage**
Sex		
Male	590	57.7
Female	432	42.3
Tumor stage		
I	573	56.1
II	283	27.7
III	166	16.2
Age (years)		
<60	564	55.2
≥60	458	44.8
BMI		
<18.5	59	5.8
18.5-24.0	553	54.1
≥24.0	410	40.1
Smoking status		
No	434	43.6
Yes	562	56.4
Histologic type		
Squamous carcinoma	269	26.3
Adenocarcinoma	593	58.0
Others	160	15.7

Note: the numbers in some groups are < 1022 due to the lack of original data. Hb: 1, Hb ≥ 120 g/L for male, ≥110 g/L for female; 2, Hb < 120 g/L for male, <110/L for female. RBC: 1, RBC ≥ 4×1012/L for male, ≥3.5 × 1012/L for female; 2, RBC < 4 × 1012/L for male, <3.5 × 1012/L for female. HCT: 1, HCT ≥ 40% for male, ≥35% for female; 2, HCT < 40% for male, <35% for female. The low group is marked 1, normal group marked 2, and high group marked 3 in each group.

**Table 2:** Univariate analysis of prognostic factors for OS of lung cancer (n = 1022).

**Variable**	β	**Wald**	**HR**	**95**%** CI**	***P* Value**
Sex (male/female, n = 1022)	-0.005	0.002	0.995	0.769-1.287	0.969
Tumor stage (I/II/III, n = 1022)					
I			1		
II	0.691	21.349	1.996	1.489-2.677	**<0.001**
III	0.993	35.920	2.699	1.951-3.735	**<0.001**
Age (<60/≥60, n = 1022)	0.189	2.147	1.208	0.938-1.556	0.143
BMI (1/2/3, n = 1022)	-0.114	1.096	0.892	0.720-1.105	0.295
Smoking (yes/no, n = 996)	-0.068	0.256	0.935	0.719-1.214	0.613
Histologic type (n = 1022)	0.150	2.382	1.162	0.960-1.407	0.123
RBC (1/2, n = 1022)	-0.642	11.106	0.526	0.361-0.768	**0.001**
Hb (1/2, n = 1022)	-0.412	6.198	0.662	0.479-0.916	**0.013**
HCT (1/2, n = 1022)	-0.575	18.464	0.563	0.433-0.731	**<0.001**
MCV (1/2/3, n = 1021)	-0.236	0.894	0.790	0.484-1.288	0.344
MCH (1/2/3, n = 1022)	-0.198	1.141	0.820	0.570-1.180	0.285

Note: BMI: body mass index; RBC: red blood cell; HB: hemoglobin; MCV: mean cell volume; MCH: mean corpuscular hemoglobin.

**Table 3:** Multivariate analysis of prognostic factors for OS of lung cancer (n = 1022).

**Variable**	β	**Wald**	**HR**	**95**%** CI**	***P* Value**
Tumor stage (I/II/III, n=1022)					
I			1		
II	0.677	20.498	1.969	1.468-2.640	<0.001
III	0.949	32.637	2.584	1.866-3.578	<0.001
HCT (1/2, n = 1022)	-0.519	14.968	0.595	0.458-0.774	<0.001

Note: the low group is marked 1 and high group marked 2.

## Data Availability

The data used to support the findings of this study have not been made available because of patients' privacy.

## References

[1] Kakimi K., Matsushita H., Murakawa T., Nakajima J. (2014). Lung cancer molecular epidemiology in China: recent trends. *Translational Lung Cancer Research*.

[2] Chen W. (2015). Cancer statistics: updated cancer burden in China. *Chinese Journal of Cancer Research*.

[3] Xie X., Yao M., Chen X. (2015). Reduced red blood cell count predicts poor survival after surgery in patients with primary liver cancer. *Medicine (United States)*.

[4] Ichinose J., Murakawa T., Kawashima M. (2016). Prognostic significance of red cell distribution width in elderly patients undergoing resection for non-small cell lung cancer. *Journal of Thoracic Disease*.

[5] Zhang F., Chen Z., Wang P., Hu X., Gao Y., He J. (2016). Combination of platelet count and mean platelet volume (COP-MPV) predicts postoperative prognosis in both resectable early and advanced stage esophageal squamous cell cancer patients. *Tumor Biology*.

[6] Chen B., Dai D., Tang H. (2016). Pretreatment Hematocrit Is Superior to Hemoglobin as a Prognostic Factor for Triple Negative Breast Cancer. *PLoS ONE*.

[7] Chen K., Chen H., Yang F., Sui X., Li X., Wang J. (2017). Validation of the Eighth Edition of the TNM Staging System for Lung Cancer in 2043 Surgically Treated Patients With Non–small-cell Lung Cancer. *Clinical Lung Cancer*.

[8] Nicholson A. G., Chansky K., Crowley J. (2016). The international association for the study of lung cancer lung cancer staging project: Proposals for the revision of the clinical and pathologic staging of small cell lung cancer in the forthcoming eighth edition of the tnm classification for lung cancer. *Journal of Thoracic Oncology*.

[9] Duan H., Zhang X., Wang F.-X. (2015). Prognostic role of neutrophil-lymphocyte ratio in operable esophageal squamous cell carcinoma. *World Journal of Gastroenterology*.

[10] Gu X., Sun S., Gao X.-S. (2016). Prognostic value of platelet to lymphocyte ratio in non-small cell lung cancer: Evidence from 3,430 patients. *Scientific Reports*.

[11] Shao N., Cai Q. (2015). High pretreatment neutrophil–lymphocyte ratio predicts recurrence and poor prognosis for combined small cell lung cancer. *Clinical and Translational Oncology*.

[12] Lefrançais E., Ortiz-Muñoz G., Caudrillier A. (2017). The lung is a site of platelet biogenesis and a reservoir for haematopoietic progenitors. *Nature*.

[13] Jagoe R. T., Goodship T. H. J., Gibson G. J. (2001). Nutritional status of patients undergoing lung cancer operations. *The Annals of Thoracic Surgery*.

[14] Kawai H., Saito Y., Suzuki Y. (2017). Gender differences in the correlation between prognosis and postoperative weight loss in patients with non-small cell lung cancer. *Interactive CardioVascular and Thoracic Surgery*.

[15] Nakagawa T., Toyazaki T., Chiba N., Ueda Y., Gotoh M. (2016). Prognostic value of body mass index and change in body weight in postoperative outcomes of lung cancer surgery. *Interactive CardioVascular and Thoracic Surgery*.

[16] Siegel I., Lin Liu T., Gleicher N. (1981). The red-cell immune system. *The Lancet*.

[17] Ibrahim U. A., Yusuf A. A., Ahmed S. G. (2016). The Pathophysiologic Basis of Anaemia in Patients with Malignant Diseases. *Gulf Journal of Oncology*.

[18] Bokemeyer C., Aapro M. S., Courdi A. (2007). EORTC guidelines for the use of erythropoietic proteins in anaemic patients with cancer: 2006 update. *European Journal of Cancer*.

